# Improving the efficiency of aerosolized insecticide testing against mosquitoes

**DOI:** 10.1038/s41598-023-33460-0

**Published:** 2023-04-18

**Authors:** Walter Fabricio Silva Martins, Emma Reid, Sean Tomlinson, George Evans, Jennie Gibson, Amy Guy, Martin Donnelly, David Weetman

**Affiliations:** 1 Laboratório de Entomologia Médica e Molecular- LEMMol, Universidade Estadual da Paraíba - UEPB, Campina Grande, Brazil; 2 Department of Vector Biology, Liverpool School of Tropical Medicine - LSTM, Liverpool, UK; 3 iiDiagnostics, Liverpool School of Tropical Medicine - LSTM, Liverpool, UK

**Keywords:** High-throughput screening, Entomology

## Abstract

Developing robust and standardised approaches for testing mosquito populations against insecticides is vital for understanding the effectiveness of new active ingredients or formulations. Methods for testing mosquito susceptibility against contact insecticides or products, such as those delivered through public health programmes, are well-established and standardised. Nevertheless, approaches for testing volatile or aerosolized insecticides used in household products can be challenging to implement efficiently. We adapted WHO guidelines for household insecticides to develop a standardised and higher-throughput methodology for testing aerosolized products in a Peet Grady test chamber (PG-chamber) using caged mosquitoes and an efficient decontamination method. The new approach was validated using insecticide resistant and susceptible *Aedes* and *Anopheles* mosquito colonies. An added feature is the inclusion of cage-facing cameras to allow real-time quantification of knockdown following insecticide exposure. The wipe-based decontamination method was highly effective for removing pyrethroids' aerosolized oil-based residues from chamber surfaces, with < 2% mortality recorded for susceptible mosquitoes tested directly on the surfaces. There was no spatial heterogeneity for knockdown or mortality of caged mosquitoes within the PG chamber. The dual-cage approach we implement yields eight-times the throughput compared to a free-flight protocol, allows simultaneous testing of different mosquito strains and effectively discriminates susceptible and resistant mosquito colonies tested side-by-side.

## Introduction

Insecticide-based interventions are the primary approach to tackle the burden of widespread vector-borne diseases, including *Aedes*-transmitted viral diseases, such as dengue, chikungunya and Zika, and malaria transmitted by *Anopheles* mosquitoes. Widespread reports of insecticide resistance indicate threats to public health anti-vector programmes^1,2^. In response alternative insecticide-based tools for public health have been explored, with increasing interest in pyrethroid-based ambient insecticides such as spatial repellents^3–5^. Herein, ambient insecticides refer to aerosolized and volatile insecticides (spatial repellents) deployed by candles, mats, emanators, sprays and coils.

To date, aerosolized insecticides have been applied primarily as household products for personal protection, with limited use in public health programmes. Nevertheless, recent studies have revealed extensive use of domestic insecticides by householders in vector-borne disease-endemic regions^6–8^. Whether or not this is motivated by nuisance-biting or disease prevention, there is a clear need for standardised and tractable approaches to screen endemic mosquito populations for susceptibility against household formulations. This is vital for predicting insecticide effectiveness against local vectors and understanding how household insecticide usage drives selection for the evolution of insecticide resistance^9^.

Improving tests for the effectiveness of ambient insecticides and deployment devices could assist in early-stage testing of multiple products against endemic vectors for utility as public health interventions. Though most ambient insecticides have pyrethroids as their active ingredient, volatile pyrethroids provide a dual mode of action, via killing activity and repellent effects, acting through alternative pathways. Pyrethroids' volatile phase interferes primarily with olfactory receptors to disrupt mosquito host-seeking behaviour and locomotion, while the residual and aerosolized droplets cause knockdown by disrupting the gating function of the voltage-gated sodium channel (*Vgsc*) target-site^5,10,11^.

Despite their widespread use and potential for further market growth, few studies have assessed the bioefficacy of spatial repellents and aerosolized formulations, especially when compared to IRS and ITNs products which act primarily as contact insecticides^2,5^. In part, this discrepancy reflects that methodologies for contact insecticides have efficient and well-established protocols, which are widely applied, such as the WHO tube test and CDC bottle assay to assess insecticide susceptibility^12,13^, and the WHO cone bioassay to evaluate insecticide treated net or residual spray effectiveness^14^.

In contrast, guidelines for testing aerosols and volatile insecticides are less well developed and have technical limitations which restrict testing capacity, such as the need for controlled environments like the Peet Grady chamber (PG-chamber)^15^. The PG-chamber is a sealed compartment (180 cm × 180 cm × 180 cm) with smooth stainless steel internal inner walls. The chamber features include extraction fans and ducts to remove aerosol vapour after each test and four large glass windows on three sides to screen mosquito knockdown.

Testing of household insecticide product bioefficacy against vector mosquitoes is expected to follow the WHO guidelines published in 2009^15^. However, the recommended approach has very low throughput, especially considering the demanding but critical decontamination step after every test, which requires thorough internal chamber washing to remove insecticide residuals. To address these limitations, we developed and validated a more standardised methodology based on the WHO guidelines, but with improved reproducibility and throughput to characterise mosquito susceptibility against aerosolized insecticides.

Our design uses a cage-based approach, which is already recommended for other ambient insecticides (coils, vaporising mats, ambient emanators and liquid vaporisers), as an alternative to the free-flying bioassay^15^, and includes three specific innovations 1. a fast wipe-based decontamination procedure, 2. a dual-cage assay to increase throughput and 3. video cameras to collect behavioural data including mosquito knockdown.

## Materials and methods

### Mosquito colonies

Four mosquito colonies with well-characterised susceptibility/resistance profiles against non-volatile contact pyrethroids (using WHO tube assays) and against volatile pyrethroids using custom plate bioassays^12,16^, were used to validate the approaches developed in this study. All colonies, two susceptible; *Ae. aegypti* (New Orleans) and *An. gambiae* (Kisumu) and two resistant; *Ae. aegypti* (Cayman) and *An. gambiae* (Tiassale) were maintained and provided by LITE (Liverpool Insect Testing Establishment). Colonies were maintained under insectary conditions: 27 ± 2 °C, 80% ± 10% relative humidity and a 12 h light: dark photoperiod.

### Aerosol insecticidal formulations

All aerosol cans used for the present work were purchased in a retail store and are household pyrethroid-based insecticide formulations. Since this work is concerned with methodology, manufacturer and product names are omitted to avoid commercial interest. The cans used are from two manufacturers and comprise household insecticides for personal protection: (A) 300 ml can with isobutane 20–30%, naphtha (petroleum) 10–20%, 1R-trans phenothrin 0.10–0.50% and prallethrin 0.10–0.50% and (B) 380 ml can with BHT (butylated hydroxytoluene) 0.005%, polyglycerol oleate 0.90%, butane and propane 30%, N—paraffin 7.5%, imiprothrin 0.040%, permethrin 0.056%, D-trans allethrin 0.108% and water 61.40%.

### Aerosol insecticidal testing

The step-by-step description of our protocol for testing aerosol insecticides is provided in Supplementary Methods 1.

**Figure 1:**
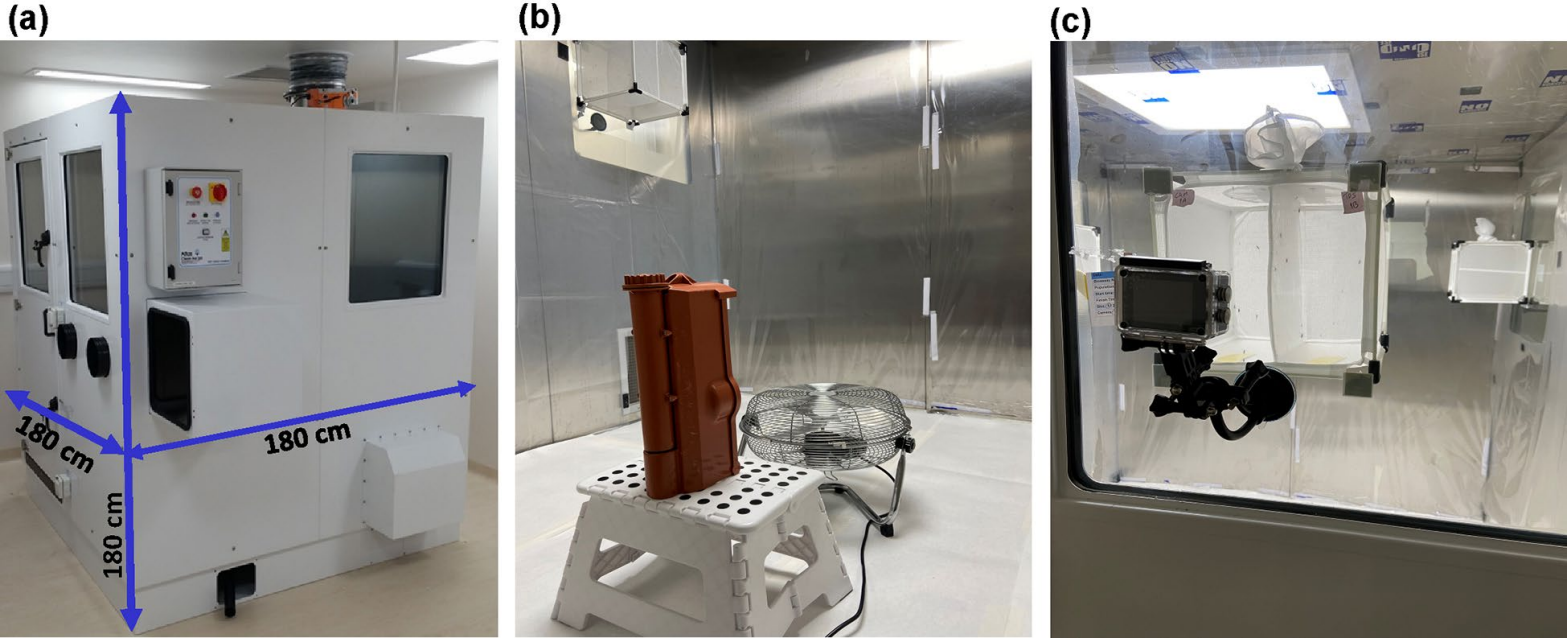
Peet-Grady chamber’s external and internal overview. (**a**) Chamber lateral profile showing glass observation windows, electrical control panel and extraction duct at the ceiling’s rear. (**b**) Set-up of the automatic aerosol dispenser and a 30-cm diameter fan in the chamber centre. (**c**) View through a chamber’s glass observation window with a camera to assist with the scoring of mosquito knockdown.

All aerosol insecticidal bioassays were performed in the PG-chamber at the Liverpool Insect Testing Establishment (LITE). The chambers were designed as outlined by WHO^15^ and manufactured by Atlas Clean Air Ltd, United Kingdom. The PG-chamber has interior measurements of 180 × 180 × 180 cm, with all internal wall panels made from polished stainless steel for easy cleaning of insecticide or solvent residues (Fig. 1a,b). After each test insecticide vapour is vented through an extractor duct located in the ceiling connected to a remote extractor fan. Screening of mosquito mortality and/or behaviour throughout testing was performed through glass observation windows at the front and chamber sides (Fig. 1a,c).

For aerosol deployment and air circulation, an automatic aerosol dispenser facing the back wall and a 30-cm diameter fan facing upwards were sited at the chamber's centre (Fig. 1b). For the fan set-up, a spirit level was used to check that the fan was horizontal to ensure even airflow circulation.

Complete personal protective equipment was worn to recover mosquitoes after aerosol testing and for chamber decontamination, including a white antistatic coverall, disposable overshoe, respirator helmet (3 M™ Versaflo™ M-206 Helmet) or safety face mask and goggles, and disposable gloves.

#### Assembly of the remote-controlled aerosol dispenser (RCAD)

Although the WHO guideline recommends the use of an automatic aerosol dispenser, specifications are not provided. Applying an automatic dispenser is crucial as manual spraying—in addition to being physically difficult in the PG-chamber—may create spatial bias and introduce variation in the duration of spraying burst.

Commercial automatic aerosol spray dispensers (Supplementary Fig. S1-A) can deploy the recommended WHO standard burst of 0.65 ± 0.10 g. However, fixed spraying burst length (single-click burst) is not feasible for testing dose–response effect of formulations. To overcome this limitation, we assembled the RCAD to operate on a switch on/off mode (Supplementary Fig. S1-B). For this purpose, we modified a commercial automatic aerosol dispenser by replacing the receiver relay with a universal wireless relay module (Supplementary Fig. S1-C), which allows us to pair the spray device to an on/off transmitter.

The RCAD reproducibility was tested for consistent deployment of aerosol insecticide within a fume hood. For this, each can was weighed before and after spraying either 3 or 5 bursts for 3 s of the aerosols described above. The discharged burst in grams from the RCAD was then compared to manual spraying using the same criteria using the same spray can. This allowed us to identify whether the primary source of variation in the burst density was the result of variation in the internal pressure of the can, the propellant concentration, or heterogenous application by the manual operator.

### Validation of the alternative approach for testing aerosol insecticides

#### Chamber and equipment decontamination

Chamber decontamination must be performed after each test, but the internal washing method recommended by the WHO guideline^15^ uses water piped through a hose. In our routine, this approach was the most time-consuming step for the bioassay set-up, so we designed and tested a wipe-based decontamination procedure.

Briefly, the wipe-based decontamination is performed by spraying 5% detergent solution (Decon 90) and surface-scrubbing using a sponge, followed by wipe-down with a stainless-steel squeegee window cleaner. Then, any detergent residue was removed by rinsing all surfaces with deionised water and once again applying the squeegee window cleaner.

To verify the effectiveness of the wipe-based approach, assuming < 20% mortality threshold for unsatisfactory decontamination as the WHO guideline^15^, after each PG–chamber decontamination, susceptible mosquitoes (Kisumu) were tested using WHO cone bioassays^14^. Six cones with 10 mosquitoes each, one per surface, were fixed onto walls of the chamber. Cone tests were performed for an exposure period of 1 h, as typically applied in WHO tube assays^12^. After exposure, mosquitoes were transferred to a holding cup and provided with 10% glucose for 24 h, after which mortality was recorded. Holding cups were kept at 27 ± 2 °C, 80 ± 10% humidity and 12:12 h photoperiod (light:dark).

Cages and other movable equipment were decontaminated by soaking in 5% Decon 90 solution for a minimum of 2 h, then rinsed thoroughly with tap water, followed by deionised water. To verify the effectiveness of cage decontamination, two cages with ten susceptible mosquitoes (Kisumu) were kept at 27 ± 2 °C, 80 ± 10% humidity and 12:12 h photoperiod (light:dark) and mortality recorded after 24h.

The removable parts of the fan were treated as above, whilst the fan blades and wireframe were cleaned with 5% Decon 90 on a sponge.

#### Dual-cage bioassay approach

To enable aerosol droplet diffusion to the cage interiors, all-around mesh cages are recommended. To provide this, we modified a 24.5 × 24.5 × 24.5 cm (650 µm mash aperture–BugDorm-4M2222 Insect Rearing Cage) using the cage’s meshed sleeve to replace the plastic on the bottom. Since PG–chamber assays are time-consuming and could be prone to locational heterogeneity, we also incorporated a split wall at the centre of the cage using the cage’s meshed sleeve (Fig. 1c). This doubles the assay throughput and allows side-by-side testing of strains for comparison (e.g., resistant and susceptible mosquitoes).

To assess whether this internal dividing wall might lead to an uneven aerosol droplet spreading within the cages, the knockdown of mosquitoes from the same colony were tested in parallel on each side. In addition to knockdown at the end of the trial, we scored knockdown every 5 min for 60 min, following direct observation at each of the four chamber's glass windows as well as from bioassay footage recorded at 60 frames per second with 1.1 × zoom using an action camera (The Xtreme I + 4 K, Campark). In some tests, at the same time as cage assays, 50 mosquitoes were released within the same chamber for direct comparison between the standard free-flying and cage-based approaches.

#### Dual-cage assay validation

Despite a fan for insecticide dispersion being applied for testing volatile products using a cage-base assay, this airflow is not required for testing free-flying mosquitoes against aerosolized insecticides following the WHO guidelines^15^. For commercial formulations, manufacturer instructions recommend a spray burst for 3–6 s, which corresponds to 5–9 g in our testing system. Such spray duration created residual droplets and the gathering of spray foam on the chamber's surface facing the aerosol deployment direction, suggesting poor homogeneity in dispersal.

We implemented a fan with a set-up as described beforehand to improve aerosol dispersion. To assess whether the fan affected the mosquito knockdown, two alternative conditions were tested: (a) fan ventilation for 1 min at the start of the assay and (b) fan ventilation for the entire 1 h of the assay. For each setting, a pairwise comparison between the free-flying and cage-based approach was performed simultaneously with the same PG-chamber using 50 free-flying resistant mosquitoes (Cayman) and four cages with 25 mosquitoes, each containing either susceptible (New Orleans) or resistant (Cayman) colonies. One cage from each colony was placed into the opposing chamber corners.

To investigate the impact of the fan's airflow disruption on the knockdown of free-flying mosquitoes, a covering was applied to the chamber's floor with a grid of 36 squares of 30 × 30 cm. The number of knocked-down mosquitoes within each square was recorded after 1 h-exposure to aerosol. Then, both free-flying and cage-confined mosquitoes were transferred to a holding cup and provided with 10% glucose for 24 h, after which mortality was recorded. Holding cups were kept at 27 ± 2 °C, 80 ± 10% humidity and 12:12 h photoperiod (light:dark).

Analysis used a binomial generalised linear model (GLM) using the IBM SPSS v26 software, with knockdown or mortality as the independent variable and airflow ventilation length and bioassay type (cage-based and free-flying mosquitoes) as factors.

## Results

### Effectiveness of decontamination

Applying our novel PG-chamber decontamination approach, we detected an overall mortality of only 1.86% (n = 268) in the insecticide-susceptible *An. gambiae* Kisumu colony in stringent 1 h-exposure cone tests (Supplementary Table S1). This demonstrates the excellent effectiveness of the method, which offers a reduction of decontamination time to 20 min compared to 1 h for the WHO methodology. No mortality was recorded in the control cages after cage decontamination based on four replicates of two cages each performed on different days, again indicating good effectiveness.

### Aerosol deployment

**Figure 2:**
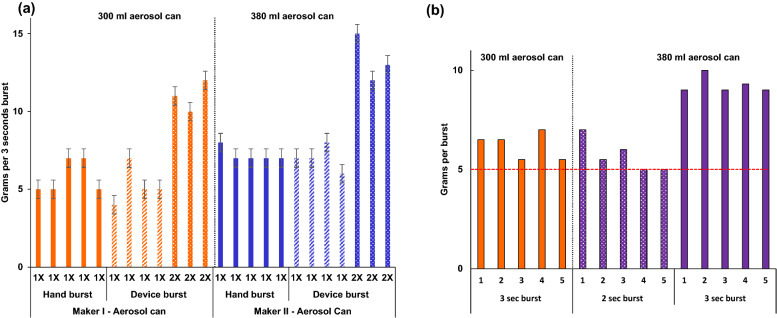
Validation and calibration of the remote-controlled aerosol dispenser (RCAD). (**a**) Comparison of spraying reproducibility within and between manual and automatic burst deployment. (**b**) RCAD burst length calibration to normalise spraying density across aerosol cans. The red dashed line is a baseline concentration to calibrate spraying burst length across variable spray can weights.

Whereas no specification for the automatic aerosol dispenser is provided by the WHO guidelines^15^, our assembled RCAD device represents a feasible design, which performs consistently and deploys a spray burst equivalent to manual spraying (fig. 2a). it is also important to bear in mind that the volume of a spray burst from a standardised spray burst duration may vary between aerosol cans due to their size as shown in fig. 2a,b. In our experimental conditions, this variation was addressed by defining a burst length to achieve 5–7 g per deployment, such as 2 and 3-s bursts for 380 ml and 300 ml can, respectively (Fig. 2b).

**Figure 3:**
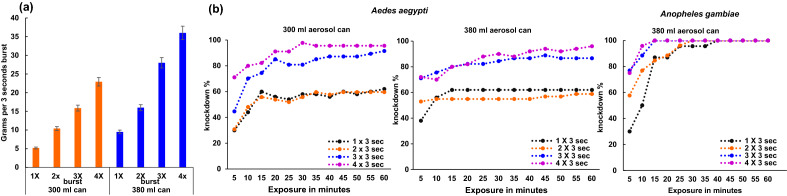
Variability in dose delivery and impacts on mosquito’s knockdown. (**a**) Change in spraying burst density deployed by automatic dispenser number of bursts, each of 3 s. (**b**) Dose–response curve of knockdown-exposure time relationships in pyrethroid-resistant colonies of *Aedes aegypti* – Cayman and *Anopheles gambiae* – Tiassale.

Based on spraying burst duration vs volume of product delivered, our aerosol dispenser also shows good accuracy in scaling the amount of aerosol deployed, at least within the tested one- to fourfold range (Fig. 3a). This feature is particularly important, as demonstrated in Fig. 3b, to facilitate the characterisation of dose–response relationships for new aerosol formulations or to establish an insecticide resistance discriminating dose.

### Dual cage-based bioassay

**Figure 4:**
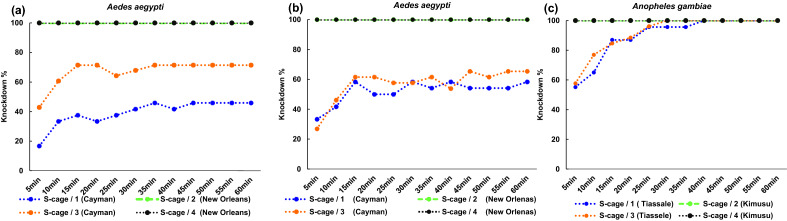
Impact of fan positioning on knockdown in relation to exposure time for *Aedes aegypti* and *Anopheles gambiae* resistant (Cayman or Tiassale) and susceptible (New Orleans or Kisumu) colonies. (**a**) fan without calibration using a spirity level (**b** and **c**) fan with spirit level calibration.

**Figure 5:**
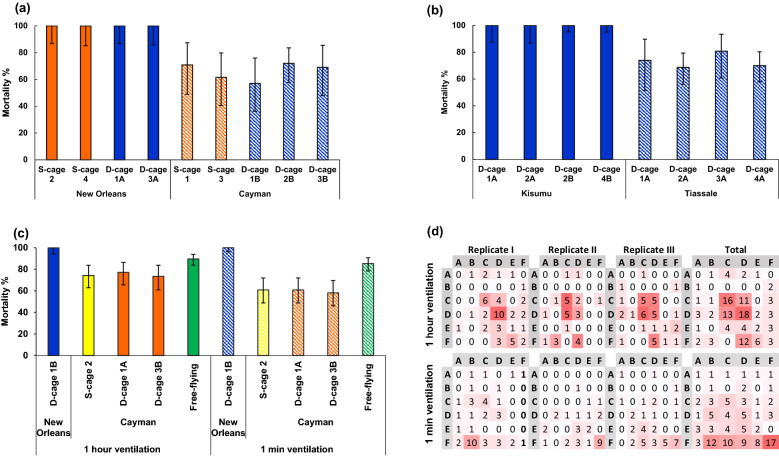
Dual-cage approach validation for screening mosquito against aerosolized insecticides. Dependence of mortality rates of susceptible and resistant (**a**) *Ae. aegypti,* (**b**) *An. gambiae* on cage structure and location in the chamber. (**c**) Impact of fan airflow duration on mortality in dual-cage and free-flying assays. (**d**) Summary of the space grid mapping of mosquito knockdown distribution in the presence/absence of fan airflow. Error bars represent 95% confidence intervals. S-cage (Standard cage) and D-cage (Dual-cage) are cages with and without an internal wall, respectively. Cage—number and letter; numbers represent the cage's clockwise location in the Peet-Grady windows and letters—A and B, dual-cage left or right half.

The dual-cage-based assay effectively discriminates resistant and susceptible tested colonies (Fig. 4a–c and Fig. 5a,b). For both susceptible strains, 100% mortality was recorded, following three replicates, with a fast knockdown effect (< 5 min) after aerosol deployment, indicating rapid contact with insecticide droplets (Fig. 4a–c). The steady knockdown rate for the *Ae. aegypti* resistant colony against each aerosol tested (Fig. 4a,b) beginning approximately 15 min after aerosol deployment suggests that neither mosquito contact with insecticide on the cage’s walls or circulation of residual droplets have a substantial impact on accumulation of knocked-down mosquitoes in the resistant strain over the 1 h-exposure period.

### Dual-cage assay validation

Our results based on the *Aedes* Cayman resistant strain revealed that an uneven fan set-up could impact aerosol bioassay reproducibility (Fig. 4a), such as for average knockdown rate (χ^2^ = 4.875, *P* = 0.03, df = 1), presumably due to heterogeneous insecticide droplet dispersion. However, this was readily corrected using a spirit level for fan horizontal orientation, following which there was no significant difference (χ^2^ = 4.671, *P* = 0.700, df = 7) in mortality between cage halves or among cages positioned next to the different observation windows (Figs. 4b,c and 5a,b). Thus, fan calibration is essential for even aerosol dispersion and resultant assay reproducibility.

Our results also revealed that fan airflow plays an important role in cage-based assays. There was a difference between the mortality rates for cage-confined mosquitoes assayed under 1-min or 1-h fan ventilation, with 27% higher mortality in the latter condition (*P* < 0.001). However, this remained 16% lower than for free-flying mosquitoes (Fig. 5c, Table 1).

**Table 1:** Binomial Generalized Linear Model (GLM) summary of mosquito mortality upon exposure to aerosolized insecticides in relation to bioassay set-up (cage-based or free-flying mosquitoes) and airflow ventilation time-length.

Parameter	B	S.E.	Wald Chi-Square	df	*P*-value
(Intercept)	0.379	0.138	7.59	1	0.006
Cage-assay / Fan—1 h	0.72	0.211	11.62	1	< 0.001
Cage-assay / Fan—1 min	0				

Parameter	B	S.E.	Wald Chi-Square	df	*P*-value
(Intercept)	1.099	0.160	47.07	1	0
Free-flying / Fan—1 min	0.777	0.294	6.98	1	0.008
Free-flying / Fan—1 h	1.052	0.302	12.12	1	< 0.001
Cage-assay / Fan—1 h	0				

In the free-flight assays, there was a notable clustering of knocked-down mosquitoes around the fan position (strata C and D within the grid in Fig. 5d) for 1-h fan ventilation. However, this was not linked with mortality, with similar rates to those for 1-min (Fig. 5c), suggesting little impact of airflow disturbance on knockdown. This indicates that either short or long-duration ventilation is suitable to overcome the problem of poor spray dispersion in unventilated conditions.

## Discussion

We show that our revised protocol for household aerosolized insecticide assessment using wipe-based decontamination, dual-cages, a remote-controlled spray device and bioassay recording with action cameras are practical alternatives to enable higher throughput than the current WHO guidelines^15^. The 20 min wipe-based approach for chamber decontamination is a major time-saver compared to the alternative (1 h per chamber in our routine). Moreover, it also diminishes user time within full personal protective equipment (e.g., respirator helmet and antistatic overalls). Furthermore, the approach minimises the likelihood of contaminating the room within which the PG chamber is housed as it produces less contaminated liquid and clothes for disposal than a full washing of the chamber. The wipe-based approach could also work well for semi-field testing room decontamination, in which a minimal furniture set-up in distinct room layouts is applied^15,17^.

The wipe-based decontamination was effective for removing residual aerosolized insecticides, as evidenced by < 2% mortality of susceptible mosquitoes (with < 20% mortality the threshold for unsatisfactory decontamination^15^) exposed to the chamber surface directly using cone tests^18^. The effectiveness of the wipe-based method could result from the scrubbing steps, which, together with the 5% detergent solution, act directly on the impregnated oil-based residuals on the chamber's surfaces. In contrast, after standard washing (detergent solution spraying, followed by a hosepipe rinse), we often recorded mortality higher than the requisite 20% threshold, which initially spurred the investigation of alternatives. The detergent solution was also effective for removing pyrethroid-based residuals from cage fabric and metal frames (based on 2 h of soaking), as indicated by zero mortality of confined susceptible mosquitoes in cleaned cages after 24 h.

A critical point addressed in our study and elsewhere^5,19^, is the challenge of standardising exposure dose for ambient insecticides among testing regimes. As shown in Fig. 2b, despite a fixed burst length, the spray volume discharged varied across aerosol cans, which likely reflects manufacturing features (e.g., variable interior pressure, propellent volume, nozzle configuration, etc.). Furthermore, the absence of a commercial remote spray device with the required feature of a flexible spraying burst-length, limits use for dose–response assessment of formulations on mosquito knockdown. Manual spraying through the chamber's door or access ports (where fitted) will likely result in varying exposure doses. Indeed, our results highlight the need for, and the importance of, reducing these sources of variation. For the *Ae. aegypti* resistant colony, we observed a relationship between aerosol dose and whether susceptibility or resistance would be concluded (Fig. 3b). Ideally, the aerosol dose should be standardised by grams delivered rather than burst length, facilitating comparison across studies.

The RCAD is an alternative for future studies to minimise the impact of technical variation. Whilst we found that standardising an aerosol burst was not feasible, we demonstrated that calibration of the burst length can approximate the mass of aerosol delivered in a repeatable manner for different aerosol cans (Figs. 2b, 3a). Such standardisations to aerosol concentration delivery within any testing chamber are vital to improving the reproducibility of results.

Our dual-cage approach could facilitate aerosolized insecticide testing routines (e.g., tracking the evolution of insecticide resistance for household formulations) by boosting test capacity by two- or eightfold compared to the standard cage and free-flying assays, respectively. Our study design provided experimental evidence, as shown in Fig. 5a,b, that the cage-based approach did not trigger heterogeneities in knockdown rate for insecticide resistance, prevent knockdown in the susceptible colonies, or delay knockdown (Fig. 4a–c).

Nevertheless, our results revealed that the fan airflow is an important feature in improving the cage assay efficiency compared to free-flight assays (Fig. 5c and Table 1). By contrast, the fan airflow has no impact on the free-flying mortality rate, although we observed a gathering of mosquito knockdown around the fan when operated for 1h (Fig. 5d). This gathering appears linked to a vacuum effect on already knocked-down mosquitoes rather than a result of airflow turbulence, which could impact survival. It is important to bear in mind that our results from free-flying are based on an assay set-up with an operating fan for aerosol dispersion, which is not applied by the current WHO guideline. A previous study testing vaporising mats^19^, also discussed the critical impact of airflow circulation on reproducibility and bias in results, evident from delayed and variable knockdown patterns when an operating fan was absent.

In our assay set-up, the fan level also impacted the bioassay results from different parts of the chamber; almost two-fold for the resistant Cayman strain (Fig. 4a). The solution to this issue is the simple use of a spirit level to ensure a straight upward direction to avoid uneven aerosol dispersal. Similarly, a previous study also attributed inconsistencies in estimated mosquito coil efficacy between laboratory and field-based testing to limited ventilation and insecticide dispersion^5^. Taken together, these insights also raise concerns about the absence of a formal guideline for semi-field study design, as often such studies^9,17,20^ have adapted a test room without standardising conditions (e.g., room size, airflow circulation and manner of aerosol deployment), as well as using a cage-based assay without ventilation to disperse the insecticide.

In our experience and that of other research groups^5,19^, the recommendations for testing aerosol assays against free-flying mosquitoes in the current guideline^15^, present substantial logistical challenges for laboratory and semi-field assessments. For instance, the free-flying approach has very low throughput (including the time-consuming recapture of surviving free-flying mosquitoes) and presents technical drawbacks, such as the impaired capacity to determine real-time knockdown.

While our validated dual-cage assay, which addresses such throughput limitations, is a feasible approach for screening mosquito susceptibility against aerosolized insecticides, it is important to highlight that the mortality recorded for cage-assayed Cayman mosquitoes was significantly lower compared to the free-flying assay (Fig. 5c, Table 1). The difference could reflect a reduced insecticide dose within the cages, as fewer aerosol droplets penetrate the mesh to the cage's interior. Further testing using fabrics with wider mesh aperture, fan airflow strength and cage layouts, such as cylindrical configurations^9,17^, could be performed to bring closer alignment between assay types.

Whether such quantitative differences observed in our present dual-cage assay set-up are important or not likely depends on the objective. When testing a new product, at least initially, the free flight assays may be preferred to allow the most precise quantitative assessment of knockdown. However, if work involves comparing mosquito strains, simultaneous testing in a more homogenised set-up presented by caged assays is likely advantageous. Moreover, we demonstrated that a hybrid approach combining both methods simultaneously is also feasible.

Remarkedly, despite recent reports of increased use of household insecticides for personal protection in countries endemic for vector-borne diseases^7,9,21^, likely due to the low availability of PG-chambers for research and additional challenges in field-based testing, there is a dearth of published studies to permit insights into issues encountered when testing household insecticides. To this end, our experiential study provides practical suggestions to address limitations in the current guidelines^15^ and to improve efficiency and throughput. Furthermore, the new features for the study design, like the use of action cameras in our routine, enhance and refine real-time data collection of the knockdown effect. Also, it facilitates screening up to eight strains in parallel and further studies of insect behaviour exposed to ambient insecticides (see Supplementary Video V1).

The dual-cage assay set-up, which allows testing multiple strains in parallel, represents a time and cost-effective platform for the study of evolving insecticide resistance to household formulations in mosquitoes from vector-borne-disease endemic regions, which could also threaten the effectiveness of public health anti-mosquito programmes.

## Conclusion

Taken together, the approaches described and validated herein facilitate reproducible testing of household insecticides at higher throughput and in a way that improves comparisons between separate strains or species, compared to the currently-advocated free-flying approach.

## Supplementary Information

Supplementary Information 1.

Supplementary Information 2.

Supplementary Information 3.

Supplementary Information 4.

Supplementary Information 5.

## Data Availability

All the data supporting the results are included in the article.
