# Supplementary material for: Improving the efficiency of aerosolized insecticide testing against mosquitoes
Source: Sci Rep. 2023 Apr 18;13:6281. doi: 10.1038/s41598-023-33460-0 (PMC10113189; doi:10.1038/s41598-023-33460-0)
Supplement: Supplementary file 2 — Supplementary Information 2. [file 41598_2023_33460_MOESM2_ESM.pdf]

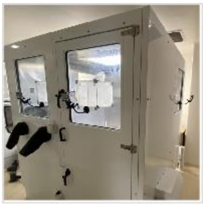

Improving the efficiency of aerosolized insecticide testing against mosquitoes.

Walter Fabricio Silva Martins[1,2], Emma Reid[2], Sean Tomlinson[2] George Evans[3], Jennie Gibson[3], Amy Guy[3], Martin Donnelly[2] David Weetman[2]

[1]Laboratório de Entomologia Médica e Molecular- LEMMol, UEPB, Campina Grande, Brasil.

[2]Department of Vector Biology, Liverpool School of Tropical Medicine - LSTM, Liverpool, United Kingdom; [3]iiDiagnostics, Liverpool of Tropical Medicine - LSTM, Liverpool, United Kingdom.

Supplementary Method 1. Step-by-step protocol for testing aerosolized insecticides in Peet-Grady chamber.

1. Mosquitoes pre-testing requirements

1 Ensure that the Peet-Grady test room is set to standard insectary conditions of 27 ± 2°C, 80%±10% RH (relative humidity) and photoperiod 12:12 hours (light: dark), as stated by the WHO.

2 Mosquitoes required for the bioassay must be sugar-fed with [M]10 % volume sugar solution and non-blood fed.

3 For each population/colony to be tested, three separate batches of 25 female mosquitoes 2-5 days old are required.

Peet-Grady set-up and decontamination

4 Cover all the Peet-Grady interior walls, floor and ceiling with a 0.75mm flexible clear plastic sheet fixed with double-sided self-adhesive Velcro to avoid damaging the chamber walls

during the scrubbing stage. Cover the chamber floors with absorbent white paper and seal

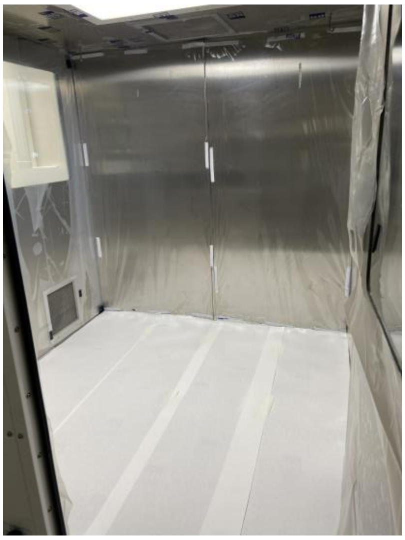

with tape.

5 Over chambers decontamination wear full PPE (Personal protective equipment)

1. Disposable white antistatic coverall
2. Disposable overshoe
3. Respirator helmet (3M™ Versaflo™ M-206 Helmet) or safety face mask and googles
4. Disposable gloves.

6 Prepare 1 L of 5% detergent solution. Dilute 50 mL of Decon 90 into 950 mL of deionized water.

Transfer the 5% detergent solution to a 1-litre multi-use sprayer.

7 Transfer 700 mL of deionized water to a multi-use sprayer.

8 For fan and foot-stool decontamination.

8.1 Remove foot-stool, flatten and place into a bucket containing [M]5 % volume of Decon 90 solution to soak for a minimum of 2 hours.

8.2 Rinse the foot-stool thoroughly with running water, and then rinse with deionized water.

8.3 Remove fan cage top to allow for easier cleaning.

8.4 Spray 2-3 bursts of the [M]5 % volume detergent solution to the hard side of a sponge and wipe the fan's blades and wireframe.

8.5 Spray deionized water on a clean sponge and wipe the fan thoroughly.

9 For automatic aerosol dispenser decontamination.

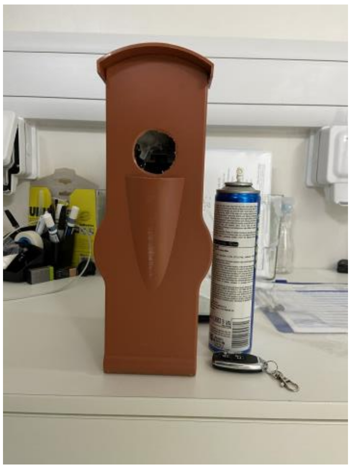

9.1 Repeat steps 8.2 and 8.3.

10 For cage decontamination.

10.1 Pull apart the cages, and follow steps 8.1 and then 8.2.

10.2 Keep cages at the set Peet Grady room conditions of 27 ± 2^{O}C, 80% ± 10% RH (relative humidity).

11 For Peet-Grady chamber decontamination

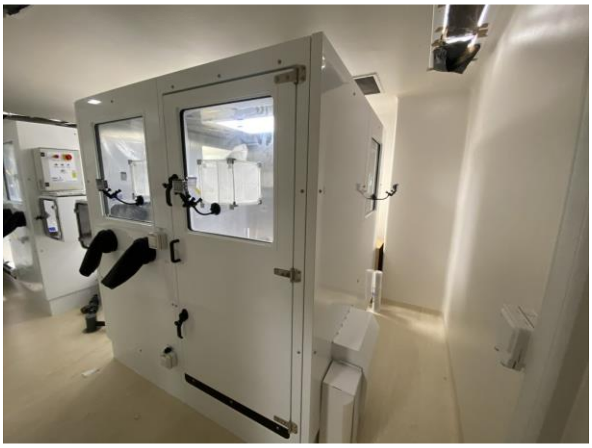

Peet-Grady Test Chamber

ATLAS CLEAN AIR LTD   Not apply

Internal measurement of 180 cm x 180 cm x 180 cm. A tight-fitting entrance door of 171cm x 80cm (guidelines suggest an approx size of 165cm x 90 cm) is positioned on one of the walls of the chamber. There is an extract duct located towards the rear of the ceiling, which is connected via a uPVC exhaust ductwork system to a remote extract fan, to remove insecticide vapour after each test.

11.1 Spray 10 mL of Decon 90 per surface across all surfaces except the floor.

11.2 Scrub all surfaces of the chamber coated with detergent using the hard side of the sponge.

11.3 Using a stainless-steel squeegee window cleaner, remove all detergent foam from the centre of the ceiling towards the walls, and then from top to bottom of each wall.

11.4 Using a multi-use sprayer, evenly spray 400 mL of deionized water across all surfaces except for the floor.

11.5 Repeat step 11.3 for the deionized water.

11.6 Remove the absorbent paper covering the floor, and shoe coverings and dispose of them in an autoclave bag.

12 Verification of chamber and cages decontamination.

12.1 Separate 6 cones with 10-female mosquitoes: at each wall, ceiling and floor is placed a cone onto surface to check the chamber is fully decontaminated. Exposed samples for 1 hour.

12.2 To assess cages decontamination, separate two cages with 25-females' mosquitoes and kept mosquitoes confined for 1 hour.

12.3 Transfer mosquitoes tested in step 12.1and 12.2 to holding cups and provide [M]10 % volume sugar-soaked cotton wool to the top of the holding cups.

Keep mosquitoes for 24 hours at 27 ± 2 °C and 80 ± 10% RH and score for mortality at 24 hours.

12.4 Repeat the decontamination steps for each chamber and/or cage if knockdown is higher than 20%.

3. Set-up of cage-based bioassay in Peet-Grady test chamber 5m

13 Set up a 30-cm diameter fan facing upwards at the centre of the chamber with an internal measurement of 180 cm x 180 cm x 180 cm. Use a spirit level to check that the fan is level. The fan may cause uneven air-flow circulation if not level.

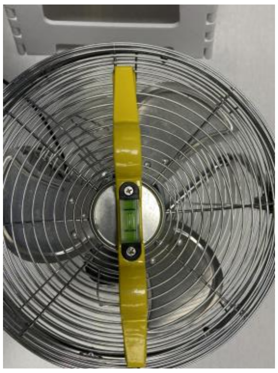

14 Quantify the aerosol spray burst in grams for each aerosol can by spraying 3 bursts of 3 seconds in a fume hood. Record the mass of each burst in grams by weighing the can before and after each burst. Spraying density may varied across can, for instance, due can total volume and interior pressure.

15 Shake the aerosol by inverting the can 3-5 times. Then, place the can inside the automatic aerosol dispenser.

16 Place a foot-stool in front of the fan 30cm apart from the fan centre. Then, stand the automatic aerosol dispenser facing the chambers' back wall. The can spray nozzle is located at 45 cm from the floor of the chamber.

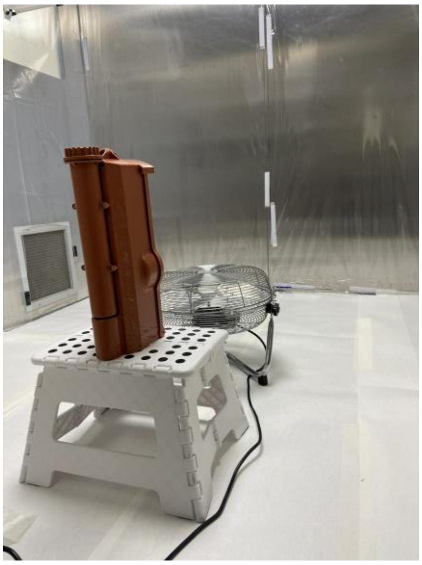

17 For real-time recording of mosquito's knockdown, in our method we have applied an action camera (The Campark Xtreme I+).
Using suction cup holders, position the action cameras outside all four chamber glass observation windows. Set each camera to record at 60 fps without audio.
.

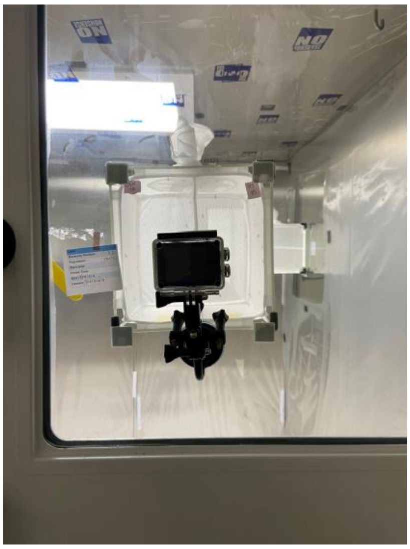

Cameras are used to assist in scoring the number of knocked-down mosquitoes (e.g. every minute for 10 minutes).

18 Transfer batches of 25 mosquitoes to four all-around mesh netting cages (20 x 20 x 20 cm).

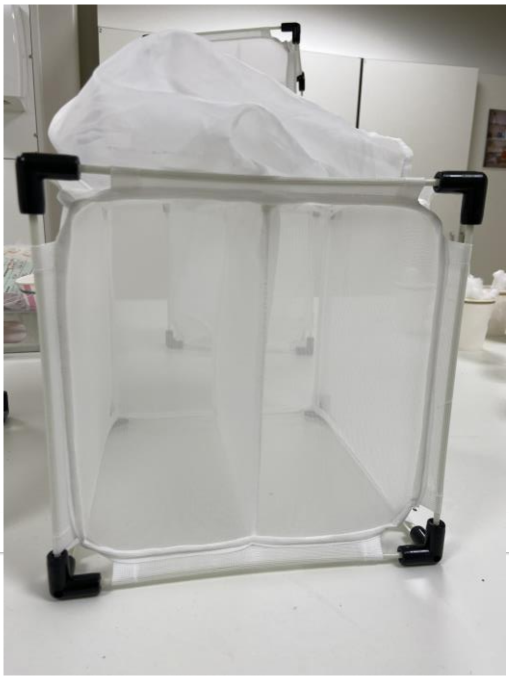

All around mesh cages are not provided by BugDorm. Bespoke the cages (0.5 mm hole opening - BugDorm-4M2222 Insect Rearing Cage) by using a meshed sleeve to replace the plastic on the bottom, as well as to set a split wall at the centre of the cage to increase the number of testing rooms.

19 Hang each cage with testing mosquitoes on the self-adhesive hooks placed at each chamber's glass observation windows. Then, switch on the fan (wind speed 4.5–5.0 m per

second) for 5
minutes for a steady airflow in the chamber.

20 Deploy aerosol insecticide bursts using the remote-controlled aerosol dispenser. The burst length for each can is established based on calibration step 14.

While the WHO protocol recommended using an automatic aerosol dispenser, further specifications or manufacture were not provided.
For our laboratory, we manufactured a remote-controlled aerosol dispenser, which allows for spraying a wide range of burst lengths. this was made using a regular remote-controlled air freshener and replacing the control with a switch-based on/off remote control. The modification was required as the original device only sprayed a 1-second burst, which was not applicable for this study design. However, the original device is suitable for the WHO spraying recommendation of 0.65 ± 0.10g.

4. Mosquitoes susceptibility profiling 1h 21m

21 For each population, perform 2-3 independent tests using batches of 25-females 2-5 days old. Between replicates, shift positions of tested populations/colonies clockwise at chambers glass observation windows.

22 Record the number of knocked-down mosquitoes every 5 minutes for 1 hour. If scoring at 1minute intervals, review the camera footage for data as scoring by eye this frequently is inaccurate. Recording data sheets are available in the annexe.

23 At the end of the test, activate the fumigation cycle at the Peet-Grady chamber control panel.

23.1 Perform the fumigation cycle for 15 minutes to purge the aerosols through the extraction vents situated in the ceiling and sides of the test chamber.

24 Wear the PPE described in step 5.

25 Transfer exposed mosquitoes using a handheld bug vacuum to holding cups and provide a [M]10 % volume sugar-soaked cotton wool pad on the top of the cups. Keep the holding cups with mosquitoes for 24 hours at 27 ± 2 °C temperature and 80 ± 10% RH and score mortality at 24 hours.

Keep the cages inside the testing chamber at all times while transferring mosquitoes to avoid contamination of the testing room.
