# Supplementary material for: Improving the efficiency of aerosolized insecticide testing against mosquitoes
Source: Sci Rep. 2023 Apr 18;13:6281. doi: 10.1038/s41598-023-33460-0 (PMC10113189; doi:10.1038/s41598-023-33460-0)
Supplement: Supplementary file 3 — Supplementary Information 3. [file 41598_2023_33460_MOESM3_ESM.pdf]

Improving the efficiency of aerosolized insecticide testing against mosquitoes

Walter Fabricio Silva Martins[1,2,*], Emma Reid[2], Sean Tomlinson[2], George Evans[3], Jennie Gibson [3], Amy Guy[3], Martin Donnelly[2], David Weetman[2].

[1]Laboratório de Entomologia Médica e Molecular- LEMMol, Universidade Estadual da Paraíba - UEPB, Campina Grande, Brasil. [2]Department of Vector Biology, Liverpool School of Tropical Medicine - LSTM, Liverpool, United Kingdom; [3] iiDiagnostics, Liverpool School of Tropical Medicine - LSTM, Liverpool, United Kingdom.

*Corresponding author: fabricio.martins@lstmed.ac.uk

Supplementary Table S1. WHO cone bioassay results for *Anopheles gambiae* susceptible mosquitoes (Kisumu) after Peet-Grady chamber wipe-based decontamination. Cone tests 1 to 4, are outcomes from four distinct testing days.

Cone Location /Replicates	Mortality – 1 h	Mortality – 24 h	Total mosquitoes	Mortality %
		**Cone test 1**		
Wall - 1/ A	0	0	9	0
Wall - 1/ B	0	0	8	0
Wall - 2 / A	0	0	9	0
Floor / A	0	0	10	0
Floor / B	1	1	10	10
Ceiling / A	0	0	9	0
Ceiling / B	0	0	9	0
**Total**	**1**	**1**	**64**	**1.56**
		**Cone test 2**		
Wall - 1	0	0	11	0
Wall - 2	0	0	9	0
Wall - 3	0	0	10	0
Wall - 4	0	0	11	0
Floor / A	0	0	11	0
Ceiling / B	0	0	8	0
**Total**	**0**	**0**	**60**	**0**
		**Cone test 3**		
Wall - 1	0	0	10	0
Wall - 2	0	0	11	0
Wall - 3	0	0	10	0
Wall - 4	0	0	11	0
Floor / A	0	0	10	0
Floor / B	0	1	10	10
Ceiling	1	1	10	10
**Total**	**1**	**2**	**72**	**2.78**
		**Cone test 4**		
Wall - 1	0	0	10	0
Wall - 2	0	0	11	0
Wall - 3	0	0	10	0
Wall - 4	0	0	11	0
Floor / A	0	0	10	0
Floor / B	0	1	10	10
Ceiling	1	1	10	20
**Total**	**1**	**2**	**72**	**4.2**
