# Supplementary material for: Improving the efficiency of aerosolized insecticide testing against mosquitoes
Source: Sci Rep. 2023 Apr 18;13:6281. doi: 10.1038/s41598-023-33460-0 (PMC10113189; doi:10.1038/s41598-023-33460-0)
Supplement: Supplementary file 5 — Supplementary Information 5. [file 41598_2023_33460_MOESM5_ESM.pdf]

Improving the efficiency of aerosolized insecticide testing against mosquitoes

Walter Fabricio Silva Martins[1,2,*], Emma Reid[2], Sean Tomlinson[2], George Evans[3], Jennie Gibson [3], Amy Guy[3], Martin Donnelly[2], David Weetman[2]

[1]Laboratório de Entomologia Médica e Molecular- LEMMol, Universidade Estadual da Paraíba - UEPB, Campina Grande, Brasil. [2]Department of Vector Biology, Liverpool School of Tropical Medicine - LSTM, Liverpool, United Kingdom; [3] iiDiagnostics, Liverpool School of Tropical Medicine - LSTM, Liverpool, United Kingdom.

*Corresponding author: fabricio.martins@lstmed.ac.uk

Supplementary Video V1. Footage showing mosquitos' knockdown after exposure to aerosolized insecticide
